# Preparation and performance evaluation of a novel temperature-resistant anionic/nonionic surfactant

**DOI:** 10.1038/s41598-024-56342-5

**Published:** 2024-03-08

**Authors:** Yongwei Duan, Yanpeng Li, Boru Chen, Chi Ai, Jun Wu

**Affiliations:** 1 College of Petroleum Engineering, Northeast Petroleum University, Daqing, 163318 China; 2 Jilin Oilfield Oil & Gas Engineering Research Institute, Songyuan, 138000 China; 3 Songyuan Gas Production Plant of Jilin Oilfield Company of PetroChina, Songyuan, 138000 China

**Keywords:** Temperature tolerance, Anionic/nonionic surfactant, Surface tension, Wettability, Resistance to adsorption, Crude oil, Energy, Chemical synthesis

## Abstract

Aiming at oil extraction from a tight reservoir, the Jilin oil field was selected as the research object of this study. Based on the molecular structures of conventional long-chain alkyl anionic surfactants, a new temperature-resistant anionic/nonionic surfactant (C_8_P_10_E_5_C) was prepared by introducing polyoxyethylene and polyoxypropylene units into double-chain alcohols. The resulting structures were characterized by Fourier transform infrared spectroscopy (FT-IR), nuclear magnetic resonance spectroscopy (^1^H-NMR), and electrospray ionization mass spectrometry (ESI–MS). Then, based on surface tension, interfacial tension, adsorption resistance, wettability, and emulsification performance tests, the performance of C_8_P_10_E_5_C was evaluated. The FT-IR, ESI–MS, and NMR spectra confirmed that C_8_P_10_E_5_C was successfully prepared. The critical micelle concentration (CMC) of C_8_P_10_E_5_C in water was 2.9510 × 10^−4^ mol/L (the corresponding mass concentration is 0.26%), and the surface tension of the aqueous C_8_P_10_E_5_C solution at this concentration was 30.5728 mN/m. At 0.3% concentration, the contact angle of the C_8_P_10_E_5_C solution was 31.4°, which is 60.75% lower than the initial contact angle. Under high-temperature conditions, C_8_P_10_E_5_C can still reduce the oil–water interfacial tension to 10^−2^ mN/m, exhibiting good temperature resistance. At 110 °C, upon adsorption to oil sand, the C_8_P_10_E_5_C solution could reduce the oil–water interfacial tension to 0.0276 mN/m, and the interfacial tension can still reach the order of 10^−2^ mN/m, indicating that C_8_P_10_E_5_C has strong anti-adsorption capability. Additionally, it has good emulsifying performance; upon forming an emulsion with crude oil, the highest drainage rate was only 50%. The forced imbibition oil recovery of C_8_P_10_E_5_C is 65.8%, which is 38.54, 24.22, and 27.25% higher than those of sodium dodecyl benzene sulfonate, alkyl polyoxyethylene ether carboxylate, and alkyl ether carboxylate, respectively.

## Introduction

As global energy requirements continue to increase, unconventional oil resources, such as tight oil, are attracting widespread attention^1–3^. Large reserves of tight oil have been found worldwide, and they have great potential for development^2,4^. A large number of micro- and nano-scale pores develop in tight reservoirs, resulting in low porosity and low permeability, complex pore throat structures, and strong heterogeneity, and production wells usually have no natural productivity^5–8^. Therefore, additional treatments, such as fracking, or other enhanced oil recovery techniques are required to achieve higher recovery rates^9,10^. Horizontal-well hydraulic fracturing is an effective technology to develop tight oil reservoirs^11–13^. By adding an imbibition solution to the fracturing fluid, crude oil can be recovered via imbibition during the shut-in process to improve the recovery efficiency of tight reservoirs^14^. Therefore, imbibition is one of the main mechanisms of enhanced oil recovery from tight reservoirs. However, because of the presence of acidic substances in crude oil, most reservoirs tend to be lipophilic^15^. Therefore, the capillary pressure of the well exerts a resistance during the imbibition process, which significantly inhibits the efficiency of imbibition^16^. Therefore, the prerequisite for reservoir imbibition is the wettability of rocks, and the stronger the hydrophilicity of the reservoir rock is, the greater the imbibition-based oil-transfer efficiency is^17,18^. Surfactants can enhance the wetting of rocks by adsorbing to them, thereby increasing the imbibition efficiency^19–22^.

However, tight reservoirs are generally buried deeper, resulting in higher reservoir temperatures. Surfactants suitable for conventional reservoirs have poor temperature resistance and are therefore unsuitable for high-temperature tight reservoirs. Consequently, there is an urgent need to develop novel temperature-resistant surfactants to improve the imbibition displacement efficiency of tight reservoirs. In this context, we developed a novel temperature-resistant anionic/nonionic surfactant. Based on the molecular structure of conventional long-chain alkyl anionic surfactants, we introduced polyoxyethylene and polyoxypropylene units to impart them with both nonionic and anionic properties, as well as better ductility, permeability, and temperature resistance. Then, we evaluated the properties of the novel temperature-resistant anionic/nonionic surfactant based on surface tension, interfacial tension, wettability, emulsification, and adsorption resistance tests. Finally, a forced imbibition experiment was conducted to evaluate the imbibition displacement efficiency.

## Experimental section

### Materials and instruments

Analytical reagent-grade isooctyl alcohol, chloroacetic acid, ethylene oxide, propylene oxide, sodium hydroxide, absolute ethyl alcohol, *n*-decane, sodium dodecyl benzene sulfonate (SDBS), alkyl polyoxyethylene ether carboxylate (APEC), and alkyl ether carboxylate (AEC) were purchased from Aladdin.

Simulated formation water was used in the experiments. The total salinity of the simulated formation water was 23,155 mg/L, and its mineral composition is listed in Table 1. Simulated formation water was used in the preparation of chemical agents used in the experiments. The dehydrated and degassed crude oil in block Q 246 of Jilin Oilfield was used as the experimental oil with a viscosity of 1.68 mP s(110 °C).

**Table 1:** Composition and properties of simulated formation water.

Ion composition	Cation (mg/L)			Anion (mg/L)				Total salinity (mg/L)
	Na^+^	Ca^+^	Mg^2+^	HCO_3_^2-^	Cl^-^	SO_4_^2-^	CO_3_^2-^	
Simulated formation water	9601	263.5	26.5	432.5	12,786.5	30.5	14.5	23,155

The following equipment were used in the study: DF-101S thermostatic heating instrument (Henan Yuhua Instrument Co., LTD.), HBYQ-2 high-temperature and high-pressure core flow test device (Huabao Petroleum Instrument Co., LTD.), vacuum rotary evaporator (Shanghai Shensheng Technology Co., LTD.), circulating water multi-purpose vacuum pump (Zhengzhou Hengyan Instrument Co., LTD.), high-temperature and high-pressure stainless steel reactor (Henan Yuhua Instrument Co., LTD.), constant-temperature water bath (Henan Yuhua Instrument Co., LTD.), DCAT21 table interface tensiometer (Dataphysics, Germany), LTD.), DCAT21 table interface tensiometer (Dataphysics, Germany), TX500C(U.S.A.) interface tensiometer (Dataphysics, Germany), OCA contact angle system (Dataphysics, Germany).

### Preparation of novel temperature-resistant anionic/nonionic surfactants

**Figure 1:**
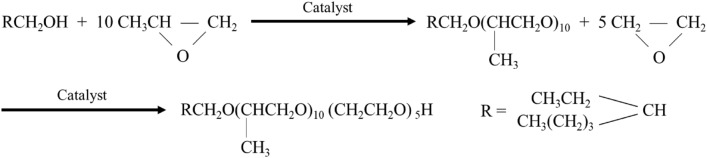
Chemical reactions involved in the synthesis of novel temperature-resistant anionic/nonionic surfactant intermediates.

**Figure 2:**
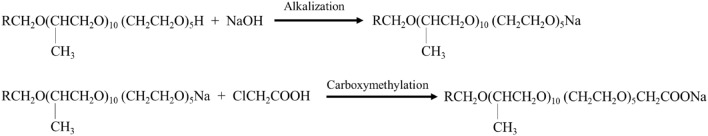
Chemical reactions involved in the synthesis of the novel temperature-resistant anionic/nonionic surfactant.

(1) Synthesis of novel temperature-resistant anionic/nonionic surfactant intermediates: double-chain alkyl alcohols (0.01 mol) and potassium hydroxide (0.015 mol) were added to a high-temperature, high-pressure reactor, and nitrogen was continuously injected into the reactor for 30 min to remove air. An explosion-proof heating box and a reactor were opened for heating. Once the temperature reached 150 ℃, a vacuum pump was connected to the reactor to reduce its pressure to − 0.1 MPa over 3 h. A metal-sealed vessel containing ethylene oxide was then heated to 40 °C in a thermostatic water bath and connected to the reactor. Thereafter, the connecting valve was opened, the reaction temperature was controlled below 170 °C until the pressure in the reactor decreased to zero, and then the reaction was terminated. Then, a metal vessel containing propylene oxide was heated to 50 °C in a thermostatic water bath and connected to the reactor. The connection valve was opened, the reaction temperature was controlled below 170 °C until the pressure in the reactor decreased to zero, and the reaction was finally terminated. When the temperature of the reactor decreased to ~ 60 °C, the reaction products were decanted to obtain double-chain alkyl polyoxyethylene/polyoxypropylene ether as a colorless liquid product. The corresponding reaction process is shown in Fig. 1.

(2) Synthesis of novel temperature-resistant anionic and nonionic surfactants: a three-necked flask was secured in an oil bath pan, and the double-branched alkyl polyoxyethylene/ polyoxypropylene ether intermediate (0.005 mol) was added to it. The temperature of the oil bath was adjusted to 70 °C, and the reactants were stirred at 700 r/min for 30 min. To ensure full contact between NaOH and the intermediate, 0.01 mol of sodium hydroxide was added to the three-necked flask in several batches, and the reaction mixture was mixed continuously at a constant temperature for 3 h. Then, 0.02 mol of chloroacetic acid was added to the three-necked flask in multiple batches, and the oil bath pan was heated to 80 °C (Note: If the temperature is extremely low, the reaction rate is slow, and if the temperature is extremely high, the probability of side reactions increases). The reaction was stopped after continuously stirring the mixture for 5 h, and a pale-yellow waxy crude product was recovered. The crude product was subsequently dried in a vacuum freeze dryer for 48 h, and anhydrous ethanol (100 mL) was added to desalinate it for improving its purity. Finally, the anionic/nonionic surfactant was obtained by removing ethanol on a rotary evaporator. The corresponding reaction process is shown in Fig. 2.

**Figure 3:**
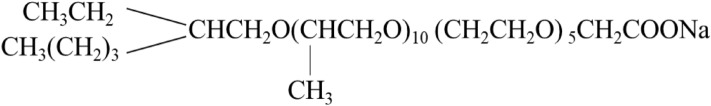
Structural formula of C_8_P_10_E_5_C.

The novel temperature-resistant anionic/nonionic surfactant was synthesized according to the aforementioned experimental methods. The structural formula of the obtained surfactant (hereafter denoted as C_8_P_10_E_5_C) is shown in Fig. 3.

### Performance evaluation of the novel temperature-resistant anionic/nonionic surfactant

#### Surface tension test

The surface tensions of a series of C_8_P_10_E_5_C solutions of different concentrations (ranging from 1 × 10^−8^ to 1 × 10^−2^ mol/L, concentration gradient of half order of magnitude) were measured using a DCAT21 interfacial tensiometer until the surface tension reached a constant value. The error in measurement was less than 0.2 mN/m.

Experimental steps: ① The quartz tank was washed thoroughly with a large quantity of water, dried with a hair dryer, soaked in a chromic acid solution for more than 5 h, and washed alternately with distilled water and ultra-pure water, and then dried. ② C_8_P_10_E_5_C solutions of different concentrations were prepared via serial dilution. ③ A platinum sheet was burned to fiery red with an alcohol lamp for 10 min for removing the surfactant and other impurities present on the surface. ④ The platinum sheet was placed vertically in the solution and then pulled out carefully, ensuring that the bottom edge of the platinum sheet was just in contact with the solution interface, and the surface tension was measured at this moment. ⑤ The surface tensions of the C_8_P_10_E_5_C solutions with different concentrations were measured successively from low concentration to high concentration.

#### Wettability test

In this experiment, the ability to change the core wettability of C_8_P_10_E_5_C solutions with different concentrations (concentration range: 1 × 10^−8^ to 1 × 10^−2^ mol/L; concentration gradient: half order of magnitude) was evaluated by the contact angle test method.

The experimental steps are as follows: ① The contact angle chamber and mineral flakes were cleaned, the contact angle chamber was vacuumed and filled with nitrogen. ② The contact angle chamber was filled with a vacuumed C_8_P_10_E_5_C solution, ensuring that the mineral flakes were completely immersed in water, and then the mineral flakes were allowed to soak at 110 ℃ for 72 h. ③ Oil droplets were injected into mineral flakes using a micro-syringe, and the shape changes of oil droplets were observed through the transparent glass of the contact angle chamber; the contact angle was measured with the help of an optical lens. ④ The concentration of the C_8_P_10_E_5_C solution was increased, and steps ①–③ were repeated until the end of the experiment.

#### Interfacial tension test

The interfacial tension between C_8_P_10_E_5_C and crude oil was measured at 90–120 ℃ (temperature gradient 20 °C) using a 0.3% C_8_P_10_E_5_C solution prepared using simulated formation water. According to the PetroChina SY/T 5370-2018 standard, the rotary drop method was used, and a rotary interface tensiometer was utilized for measurements. The oil:water volume ratio was approximately 1:200, the rotation speed was 5000 r/min, and the measurement was performed until the steady-state value of the dynamic interfacial tension was reached, that is, when the value of the interfacial tension changed by less than 1% during 30 min.

#### Adsorption resistance test

Treatment of oil sands: The oil sand was suspended in petroleum ether, stirred with a glass rod, and soaked for approximately 10 min. Then, the solvent (petroleum ether) was removed, and the process was repeated two to three times until no oil remained on the surface of the oil sand. After drying (2 h), a small amount of the oil sand was placed in hot water; the absence of an oil slick indicated a thorough cleaning.

Experimental steps: ① The interfacial tension of the C_8_P_10_E_5_C solution was measured before adsorption to oil sand. ② The oil sand and a C_8_P_10_E_5_C solution were taken in a ground conical bottle with a stopper at a 1:10 solid/liquid ratio. ③ The conical bottle was subjected to constant-temperature oscillation in a water bath at 110 °C for 48 h. ④ The solution in the conical flask was shaken uniformly and transferred to a centrifuge tube. After centrifugation for 30 min, the supernatant was collected and its interfacial tension was measured.

#### Evaluation of the emulsifying property

The bottle test method was used to determine the stability of the emulsion: a 0.3% C_8_P_10_E_5_C solution and crude oil were maintained at 110 °C for 30 min, and then 10 g of the solution was taken according to the ratio of oil to water (1:1 mass ratio). The mixture was poured into a 25 mL stoppered test tube in the order of water first and then oil, the test tube was plugged with the stopper, and the contents were shaken vigorously by hand 100 times to ensure that it was completely emulsified. Once the desired emulsion was obtained, it was kept warm in a thermotank, and record the amount of water produced at different times.

#### Forced imbibition experiment

**Figure 4:**
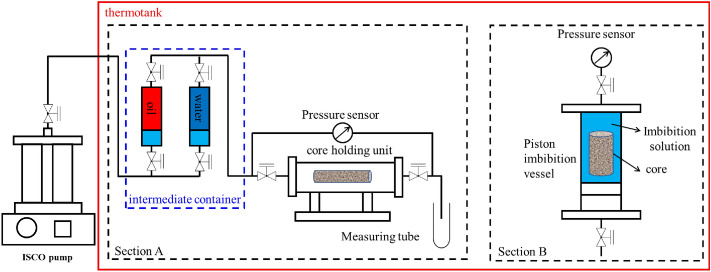
Experimental apparatus of forced imbibition.

The forced imbibition experiment was conducted to evaluate the imbibition displacement efficiency of C_8_P_10_E_5_C by simulating the imbibition process of the imbibition liquid under the formation pressure (21.4 MPa) and temperature (110 °C) in a piston imbibition vessel. The experimental device is shown in Fig. 4.

Experimental procedure: ① The core was placed in an oven at 110 °C for 4 h and then cooled to room temperature. Its weight was recorded as m_1_. Then, it was dried further at 110 °C for 30 min, cooled to room temperature, and the weight was recorded as m_2_. When m_2_–m_1_ was less than 0.0005 g, the core was considered to be sufficiently dry, and the dry weight of the core was calculated as m_3_ = (m_1_ + m_2_)/2. ② The experimental apparatus was first connected to part A, and the rock sample was vacuumed for 4 h and then saturated with the simulated formation water. The core was removed and weighed (m_4_); the saturated water mass of the core was calculated as W_1_ = m_4_ − m_3_. ③ The core was placed in a gripper and aged for 24 h. The oil was injected at a rate of 0.02 mL/min until the water content in the produced liquid was 0. The volume of water in the produced liquid, V_1_ (volume of saturated oil in the core), was recorded. In addition, the bound water mass was determined as W_2_ = m_4_ − m_3_ − V_1_. ④ The core was removed, placed in a beaker with experimental oil for 2 h, the surface oil slick was removed, and it was quickly weighed for three consecutive times at different positions of the balance, and the average mass was recorded as W_3_; the weight of saturated oil was calculated as W_4_ = W_3_ − m_3_ − W_2_. Then, the experimental device was connected to part B, and different imbibition solutions were added according to the experimental scheme in Table 2. The rock sample was placed in the piston imbibition vessel, pressurized to the formation pressure, and the valves at both ends of the intermediate vessel were closed. The intermediate container was then placed in the incubator, removed after 24 h, cooled to room temperature, the core was retrieved, the surface floating oil and water were removed, and it was quickly weighed at different positions of the balance three consecutive times; the average mass of three weights was recorded as W_5_. The volume of the oil produced by forced imbibition was calculated as V_2_ = (W_5_ − W_3_)/(1 − W_4_/V_1_), and the imbibition oil recovery is given by V_2_/V_1_.

**Table 2:** Reverse imbibition experiment scheme.

Core number	Surfactant type	Length/(mm)	Diameter/(mm)	Permeability/(mD)	Porosity/(%)
L-1	C_8_P_10_E_5_C	61.2	25.0	0.1432	15.40
L-2	SDBS	62.6	25.0	0.1514	14.8
L-3	APEC	64.1	25.0	0.1365	15.11
L-4	AEC	63.5	25.0	0.1129	14.57

## Results and discussion

### Structural characterization of C_8_P_10_E_5_C

**Figure 5:**
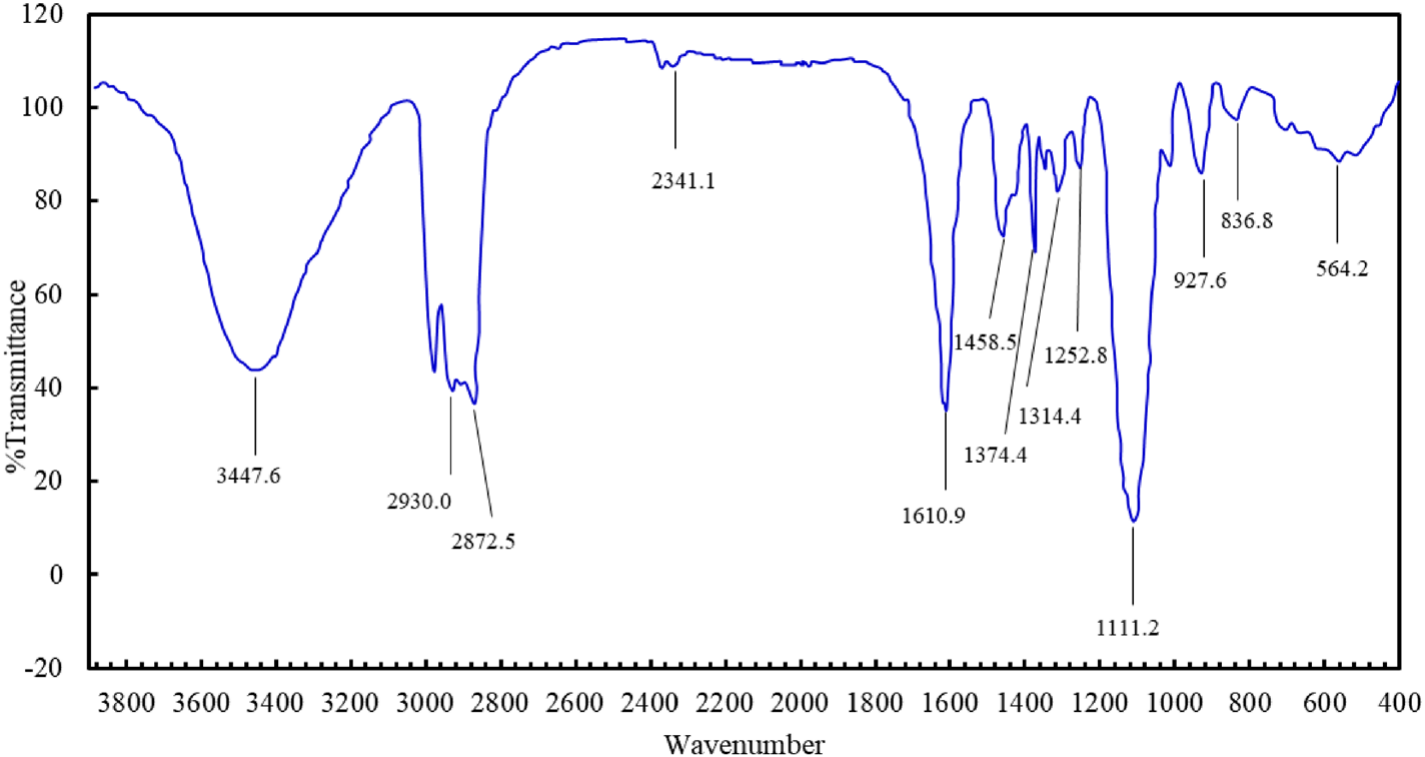
Fourier transform infrared (FT-IR) spectrum of C_8_P_10_E_5_C.

**Figure 6:**
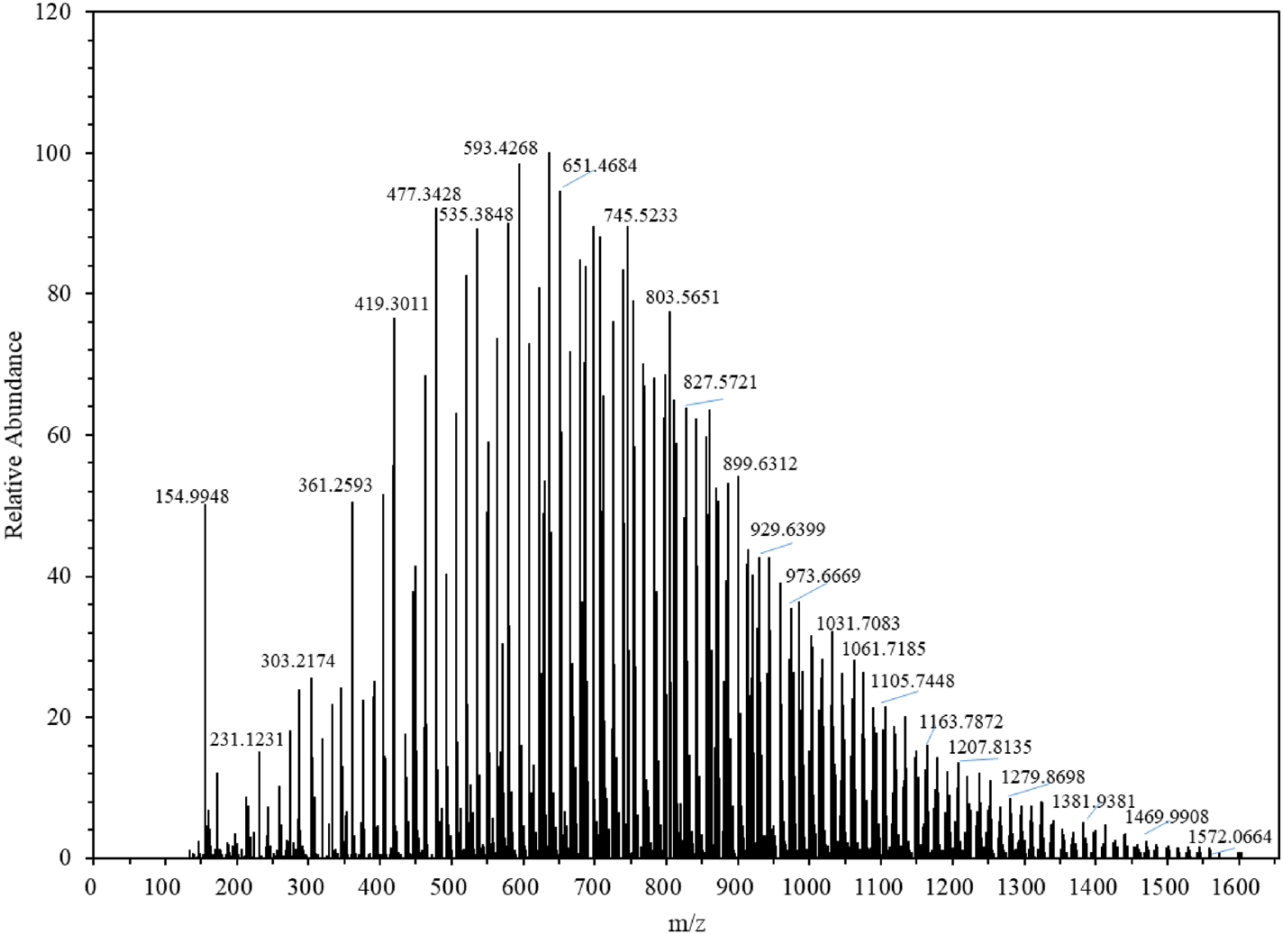
Electrospray ionization mass spectrometry (ESI–MS) of C_8_P_10_E_5_C.

**Figure 7:**
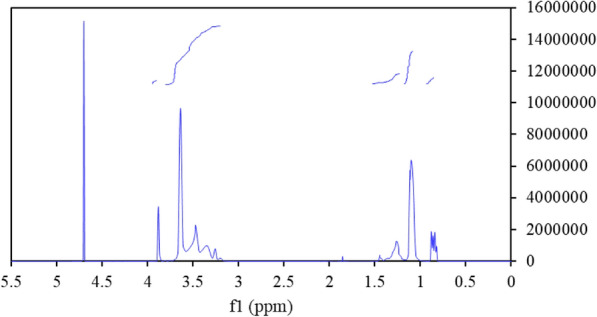
^1^H-NMR spectrum of C_8_P_10_E_5_C.

The structure of the C_8_P_10_E_5_C surfactant was analyzed by Fourier transform infrared (FT-IR) spectroscopy, electrospray ionization mass spectrometry (ESI–MS), and nuclear magnetic resonance (^1^H-NMR). The results are shown in Figs. 5, 6, and 7. As shown in Fig. 5, the FT-IR absorption peak at 3447 cm^−1^ was attributed to the hydroxyl or amino group (OH or NH) of C_8_P_10_E_5_C, and the absorption peaks at 2930 and 2872 cm^−1^ were assigned to the stretching vibrations of its methyl or methylene (–CH_3_ or –CH_2_) groups. The absorption peak at 1610 cm^−1^ was attributed to the carbonyl group (COO^−^) and that at 1458 cm^−1^ was assigned to methylene (–CH_2_) deformational vibrations. Further, the absorption peak at 1374 cm^−1^ corresponds to the deformational vibrations of the methyl groups (–CH_3_) and those at 1314 and 1252 cm^−1^ are due to the asymmetric stretching vibrations of the carbonyl group (COO^−^). The absorption peak at 1111 cm^−1^ corresponds to the vibrations of the ether bond (–CH_2_OCH_2_-), and the absorption peaks at 927 and 836 cm^−1^ are due to the vibrations of the polyether backbone (–CH_2_CH_2_O). Thus, the molecular chains contained all the designed groups and met the expected results. Therefore, the results of FT-IR spectroscopy preliminarily indicate that the target synthetic product, C_8_P_10_E_5_C, was formed.

As shown in Fig. 6, the molecular ion peaks in the mass spectrum are 303, 361, 419, 477, 535, 593, 651, 745, 803, 827, 899, 929, 973, 1031, 1061, 1105, 1163, 1207, 1279, etc. The multiple sets of data with peak differences of 44 and 58 indicate the presence of compounds containing both polyoxyethylene and polyoxypropylene units in the sample. Thus, ESI–MS further confirmed that the targeted synthetic product, C_8_P_10_E_5_C, was successfully obtained.

As shown in Fig. 7, the chemical shift of 0.83 ppm corresponds to the protons of –CH_3_, the chemical shift of 1.07 ppm corresponds to the protons of CH_3_CHCH_2_O, the chemical shift of 1.27 ppm corresponds to the protons of –CH_2_ groups, the chemical shifts of 3.67, 3.50, 3.41 and 3.30 ppm correspond to the protons of CH_3_CHCH_2_O and CH_2_O moieties, and the chemical shift of 3.92 ppm corresponds to the protons of the CH_2_COO^−^ unit. The proton peaks corresponding to each unit were observed in the ^1^H-NMR spectra of C_8_P_10_E_5_C. Combined with the results of FT-IR spectroscopy and ESI–MS, these results confirm that the product was the new anionic/nonionic surfactant, C_8_P_10_E_5_C.

### Evaluation of the performance of C_8_P_10_E_5_C as a surfactant

#### Surface tension

**Figure 8:**
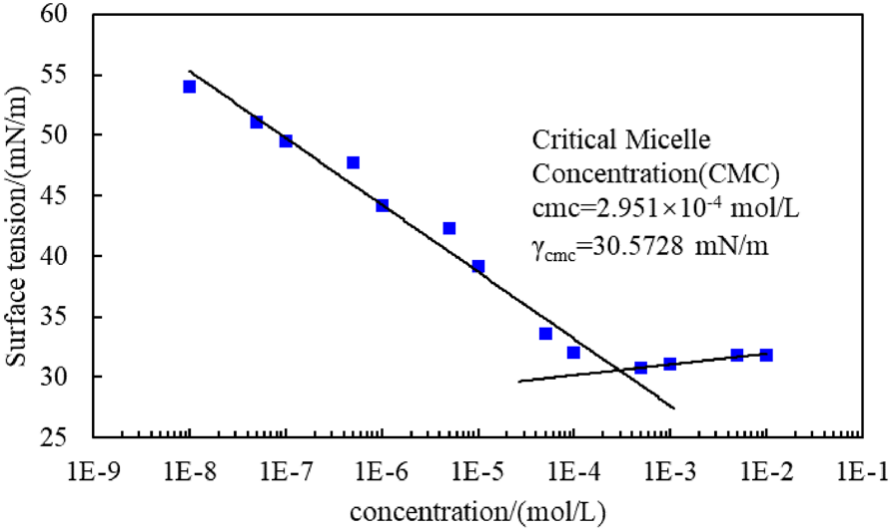
Surface tension vs. the concentration of the C_8_P_10_E_5_C solution.

The surface tension results for C_8_P_10_E_5_C solutions of different concentrations are shown in Fig. 8. Initially, as the concentration of the C_8_P_10_E_5_C solution increased, its surface tension gradually decreased, and then the surface tension started to increase. The concentration corresponding to the shift in the change trend is the critical micelle concentration (CMC) of the C_8_P_10_E_5_C surfactant. The inflexion point of surface tension was determined using tangential fitting. Thus, the CMC value of C_8_P_10_E_5_C was determined to be 2.951 × 10^−4^ mol/L (the corresponding mass concentration is 0.26%). The surface tension of the solution at this concentration was 30.5728 mN/m. Thus, the synthesized C_8_P_10_E_5_C surfactant exhibited satisfactory surface activity.

#### Wettability test

**Figure 9:**
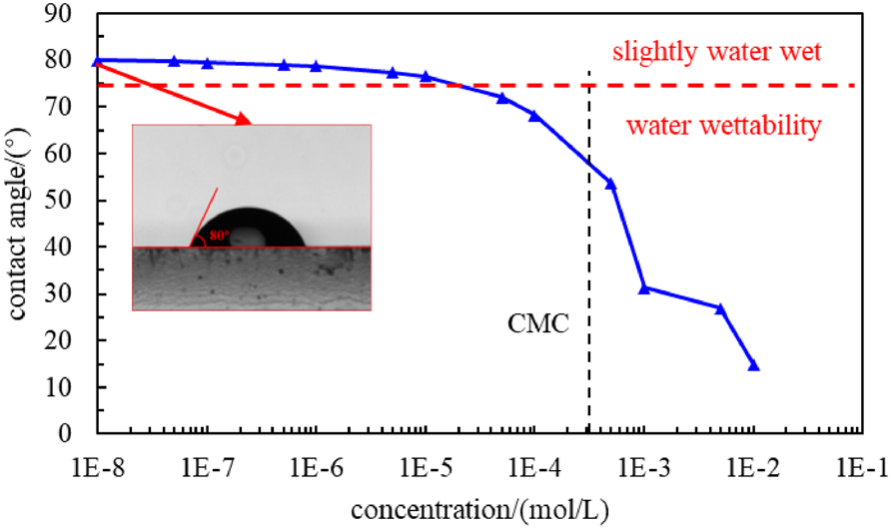
Variation in the contact angle of the core with a change in concentration of the C_8_P_10_E_5_C solution.

Figure 9 shows the variation in the contact angle of the core with a change in the concentration of the C_8_P_10_E_5_C solution. When the concentration of C_8_P_10_E_5_C was less than 1 × 10^−5^ mol/L, the contact angle was greater than 75°, and the core was slightly wetted with water. When the concentration exceeded this range, the contact angle decreased slightly. However, when the CMC was reached, the contact angle decreased significantly. When the concentration of the C_8_P_10_E_5_C solution was 0.3%, the contact angle was 31.4°, which is 60.75% lower than the initial contact angle of the core, and the core exhibited water wettability. In summary, when the concentration of the C_8_P_10_E_5_C solution was higher than its CMC, the wettability improvement effect was better. Therefore, a C_8_P_10_E_5_C solution with a concentration higher than the CMC was selected for subsequent experiments to ensure better surface activity and improved wettability. To minimize the development costs, a 0.3% C_8_P_10_E_5_C solution was selected as the imbibition displacement agent.

#### Interfacial tension test

**Figure 10:**
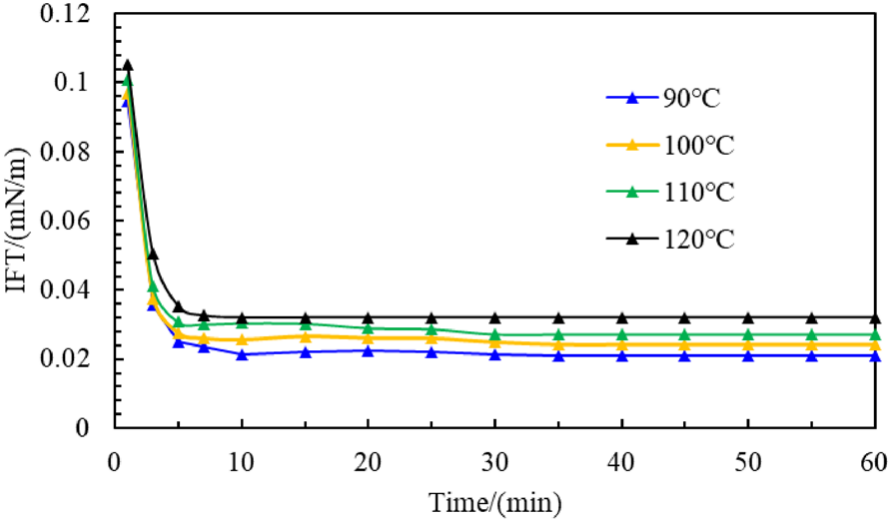
Interfacial tension curves of a C_8_P_10_E_5_C solution (0.3%) and crude oil at different temperatures.

The test results of the interfacial tension between the C_8_P_10_E_5_C surfactant solution with a concentration of 0.3% and crude oil at different temperatures are shown in Fig. 10. The interfacial tension first decreased sharply with time, then decreased slowly, and finally stabilized. This is because the composition of the crude oil is complex. The initial oil–water interfacial tension is high. After a certain duration, the surfactant molecules emulsify the oil surface and penetrate the oil body, organizing at the oil–water interface to reduce the interfacial tension between them. The interfacial tension between the C_8_P_10_E_5_C solution and crude oil stabilized to 0.0211, 0.0243, 0.0273, and 0.0321 mN/m at 90, 100, 110, and 120 °C, respectively. At high temperatures, C_8_P_10_E_5_C could still decrease the oil–water interfacial tension to 10^−2^ mN/m. Thus, the capability of C_8_P_10_E_5_C in reducing the oil–water interfacial tension was not affected by the temperature, which indicates its good temperature resistance.

#### Adsorption property test

**Figure 11:**
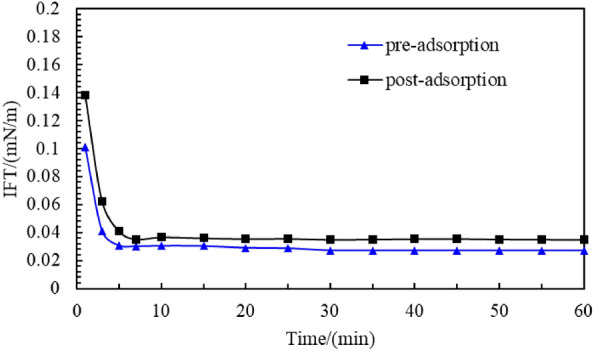
Interfacial tension curve of C_8_P_10_E_5_C before and after adsorption.

The change in the interfacial tension between the C_8_P_10_E_5_C solution (0.3% concentration) and crude oil before and after adsorption onto oil sand is shown in Fig. 11. Both before and after adsorption on the oil sand, the interfacial tension of the C_8_P_10_E_5_C solution first decreased sharply with increasing time, then decreased slowly, and finally became stable. Before adsorption, the interfacial tension between the C_8_P_10_E_5_C solution and crude oil was 0.0273 mN/m. After adsorption, the surfactant concentration decreased, and its effect of reducing the interfacial tension was weakened. Therefore, after adsorption, the interfacial tension between the C_8_P_10_E_5_C solution and crude oil was 0.0348 mN/m, 27.56% higher than that before adsorption, but it could still reach the order of 10^−2^ mN/m. This result indicates that C_8_P_10_E_5_C has strong anti-adsorption capability.

#### Emulsifying property test

**Figure 12:**
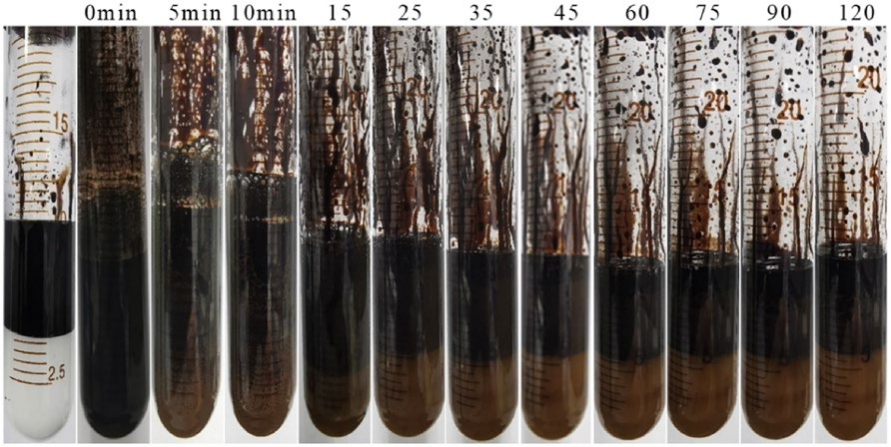
Crude oil − water separation after emulsification using C_8_P_10_E_5_C.

Emulsification is an important mechanism by which surfactants enhance oil recovery. After the surfactant comes into contact with the crude oil, emulsification breaks the large oil droplets into smaller ones, allowing the crude oil to pass through the smaller pores and thus improving the oil-washing efficiency. The stability of the emulsion was observed using the bottle test method, and the results are shown in Fig. 12. As shown, with the prolongation of the placement time, the oil − water two-phase separation became evident, indicating that the synthesized C_8_P_10_E_5_C had an excellent emulsifying capability.

**Figure 13:**
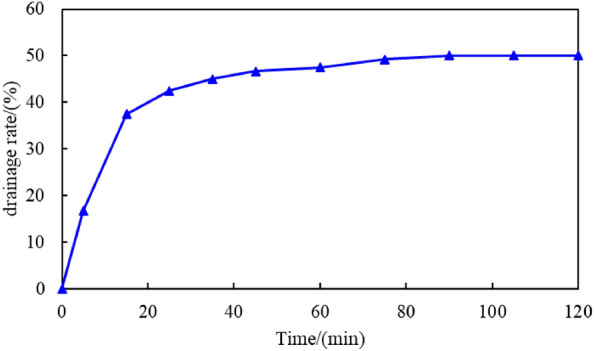
Drainage rate of the C_8_P_10_E_5_C emulsion system.

The stability of the emulsion formed using the C_8_P_10_E_5_C solution and crude oil was evaluated based on the drainage rate. The C_8_P_10_E_5_C solution was emulsified with crude oil to form an emulsion, and the drainage of the emulsion at different time points was evaluated (see Fig. 13). As shown, with the increase in time, the drainage first increased sharply to a certain value, then slowly to the highest value, and then tended to be stable. The highest drainage rate was 50%. The maximum drainage rate is small, indicating that the emulsion formed by the emulsification of the C_8_P_10_E_5_C solution with crude oil was stable, and the emulsification performance of C_8_P_10_E_5_C was strong.

#### Assessment of the forced imbibition efficiency

**Figure 14:**
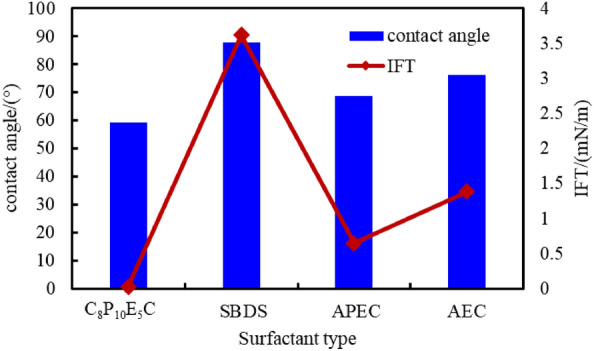
Test results of interfacial tension and contact angle for different types of surfactants.

To evaluate the imbibition efficiency of C_8_P_10_E_5_C under high-temperature formation, its imbibition efficiency was compared with those of three other surfactants (SBDS, APEC, and AEC). The test results of the oil−water interfacial tension and contact angle of the four surfactants under the same conditions are shown in Fig. 14. Among the four surfactants, C_8_P_10_E_5_C exhibited the best effect in reducing the interfacial tension between oil and water and improving the wettability of the oil sand under the formation temperature conditions, indicating that it has strong high-temperature resistance.

A forced imbibition experiment was conducted to evaluate the imbibition oil recovery of different surfactants, and the imbibition oil recovery calculation results are shown in Table 3. As shown, the forced imbibition oil recovery values of C_8_P_10_E_5_C, SBDS, APEC, and AEC are 65.80, 27.26, 41.57, and 38.55%, respectively. According to these results, the forced imbibition oil recovery of C_8_P_10_E_5_C is 38.54, 24.22, and 27.25% higher than those of SBDS, APEC, and AEC, respectively. Thus, the efficiency of the new temperature-resistant anionic/nonionic surfactant in forced imbibition oil recovery from tight reservoir is significantly better than those of conventional anionic and nonionic surfactants. Therefore, compared with conventional anionic and nonionic surfactants, the new temperature-resistant anionic/nonionic surfactant is more suitable for enhanced oil recovery by imbibition from tight reservoirs.

**Table 3:** Calculation results of the forced imbibition oil recovery achieved using different surfactants.

Core number	Core saturated water			Core saturated oil				Evaluation of imbibition efficiency		
	m_3_/g	m_4_/g	W_1_/g	V_1_/mL	W_2_/g	W_3_/g	W_4_/g	W_5_/g	V_2_/mL	Imbibition recovery/%
L-1	77.1862	81.8124	4.6263	3.1463	1.4800	81.3253	2.6592	81.6458	2.0702	65.80
L-2	76.7850	81.3342	4.5492	2.8842	1.6650	80.7145	2.2645	80.8834	0.7861	27.26
L-3	77.0346	81.7912	4.7567	3.0014	1.7553	81.1338	2.3440	81.4071	1.2478	41.57
L-4	77.8915	82.4342	4.5427	3.9842	0.5585	81.8145	3.3645	82.0534	1.5359	38.55

## Discussion

Based on the molecular structures of conventional long-chain alkyl anionic surfactants, a new temperature-resistant anionic/nonionic surfactant (C_8_P_10_E_5_C) was synthesized by introducing polyoxyethylene and polyoxypropylene units into double-chain alcohols. The FT-IR and mass spectra of C_8_P_10_E_5_C confirmed that the molecular chain of C_8_P_10_E_5_C contained all the designed groups and that the sample consisted of compounds containing both polyoxyethylene and polyoxypropylene units. In addition, the corresponding proton peaks of each unit were observed in the ^1^H-NMR spectrum of the synthetic product, C_8_P_10_E_5_C, consistent with the expected results. Thus, the final synthesized product was confirmed to be C_8_P_10_E_5_C.

The CMC of C_8_P_10_E_5_C was 2.951 × 10^−4^ mol/L, and the surface tension of the C_8_P_10_E_5_C solution at this concentration was 30.5728 mN/m. When the concentration of the C_8_P_10_E_5_C solution was higher than the CMC, the wettability improvement was better. When the concentration was 0.3%, the contact angle of the solution on the core was 31.4°, which is 60.75% lower than the initial contact angle of the core. Under high-temperature conditions, C_8_P_10_E_5_C could still reduce the oil–water interfacial tension to 10^−2^ mN/m. Thus, the capability of C_8_P_10_E_5_C in reducing the oil–water interfacial tension was not affected by the temperature, indicating its good temperature resistance. At 110 °C, the C_8_P_10_E_5_C solution adsorbed to oil sand could reduce the oil–water interfacial tension to 0.0276 mN/m, and it could still reach the order of 10^−2^ mN/m. This result indicates that the C_8_P_10_E_5_C has strong anti-adsorption ability. In addition, it has good emulsifying performance; when an emulsion was formed with crude oil, the highest drainage rate was only 50%.

The forced imbibition oil recovery of C_8_P_10_E_5_C was 38.54, 24.22, and 27.25% higher than those of SBDS, APEC, and AEC, respectively. Compared to conventional anionic and nonionic surfactants, the new temperature-resistant anionic/nonionic surfactant is more suitable for enhanced oil recovery by imbibition from tight reservoirs.

## Data Availability

The datasets used and/or analyzed during the current study available from the corresponding author on reasonable request.
